# Human FoxP Transcription Factors as Tractable Models of the Evolution and Functional Outcomes of Three-Dimensional Domain Swapping

**DOI:** 10.3390/ijms221910296

**Published:** 2021-09-24

**Authors:** Pablo Villalobos, César A. Ramírez-Sarmiento, Jorge Babul, Exequiel Medina

**Affiliations:** 1 Departamento de Biología, Facultad de Ciencias, Universidad de Chile, Las Palmeras 3425, Casilla 653, Santiago 7800003, Chile; pavillalobos@u.uchile.cl (P.V.); jbabul@uchile.cl (J.B.); 2 Institute for Biological and Medical Engineering, Schools of Engineering, Medicine and Biological Sciences, Pontificia Universidad Católica de Chile, Santiago 7820436, Chile; cesar.ramirez@uc.cl; 3 ANID—Millennium Science Initiative Program, Millennium Institute for Integrative Biology (iBio), Santiago 8331150, Chile

**Keywords:** domain swapping, protein flexibility, FoxP subfamily, protein evolution, local unfolding, high-resolution biophysics

## Abstract

The association of two or more proteins to adopt a quaternary complex is one of the most widespread mechanisms by which protein function is modulated. In this scenario, three-dimensional domain swapping (3D-DS) constitutes one plausible pathway for the evolution of protein oligomerization that exploits readily available intramolecular contacts to be established in an intermolecular fashion. However, analysis of the oligomerization kinetics and thermodynamics of most extant 3D-DS proteins shows its dependence on protein unfolding, obscuring the elucidation of the emergence of 3D-DS during evolution, its occurrence under physiological conditions, and its biological relevance. Here, we describe the human FoxP subfamily of transcription factors as a feasible model to study the evolution of 3D-DS, due to their significantly faster dissociation and dimerization kinetics and lower dissociation constants in comparison to most 3D-DS models. Through the biophysical and functional characterization of FoxP proteins, relevant structural aspects highlighting the evolutionary adaptations of these proteins to enable efficient 3D-DS have been ascertained. Most biophysical studies on FoxP suggest that the dynamics of the polypeptide chain are crucial to decrease the energy barrier of 3D-DS, enabling its fast oligomerization under physiological conditions. Moreover, comparison of biophysical parameters between human FoxP proteins in the context of their minute sequence differences suggests differential evolutionary strategies to favor homoassociation and presages the possibility of heteroassociations, with direct impacts in their gene regulation function.

## 1. Introduction

Oligomerization into quaternary structures is one of the most determinant aspects by which proteins modulate or even gain new functions [1,2]. Fundamental cellular processes such as signal transduction, enzyme activity, gene expression, and membrane transport are regulated by changes in quaternary complexes [3].

An evolutionary advantage of oligomerization is that it allows the formation of large macromolecular complexes with additional functional sites in comparison to isolated monomers, all without increasing genome size [4,5]. Concomitantly, several protein structure databases convey that homooligomers are dominant over monomers [1,6], emphasizing the relevance of understanding the evolution of protein–protein interactions and their role in both structural and functional diversification in all organisms.

**Figure 1:**
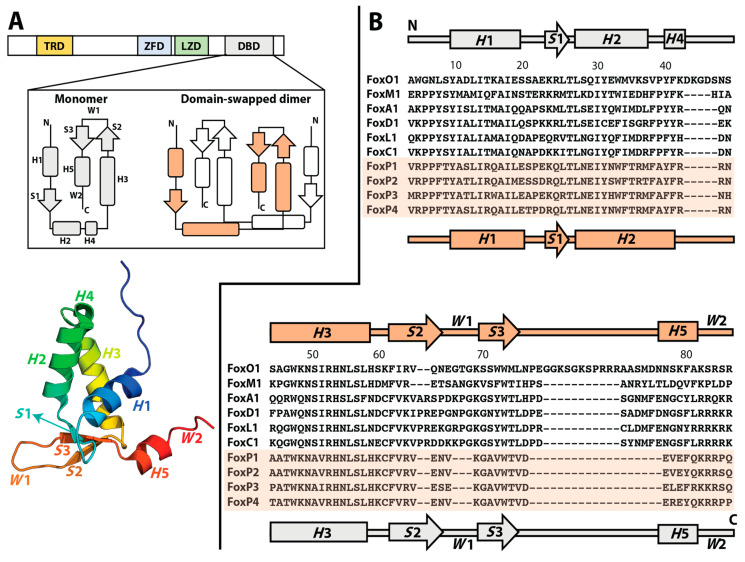
Three-dimensional domain swapping (3D-DS) in FoxP proteins. (**A**) Human FoxP proteins are constituted by a trans repressor domain (TRD), a zinc finger domain (ZFD), a leucine zipper domain (LZD), and a DNA-binding domain (DBD). Evolutionary changes in the latter enable FoxP proteins to associate into domain-swapped dimers. The secondary structure topology of the DBD is shown for both monomeric and dimeric FoxP. All secondary structure elements are colored and highlighted in the three-dimensional structure of a monomeric mutant of the DBD of FoxP1 (PDB 2KIU). (**B**) Sequence comparison of the DBD from different Fox subfamilies, showing the secondary structure topology for the monomeric members in gray and for the dimeric FoxP proteins in orange. Note that helices *H2* and *H4* in the monomer merge into a single helix *H2* in the domain-swapped dimer. FoxP sequences are highlighted in light orange.

The emergence of oligomers in nature starting from ancestral monomers has been largely explained by the accumulation of random mutations on the surface of the monomers throughout evolution, which generates an optimized interface that enables protein–protein interactions [7,8]. These mutations allow the stabilization of hydrophobic interactions, salt bridges, and hydrogens bonds, which are the main interactions characterized as fundamental in enabling the stabilization of protein–protein interfaces during oligomer evolution [7,8]. However, three-dimensional domain swapping (3D-DS) has been proposed as an alternative and ancient mechanism for the evolution of protein oligomerization that does not require an optimized interaction surface [9]. In this mechanism, two (or more) protein chains exchange identical elements of their structures to form an intertwined oligomer [10] (Figure 1A).

In order to reach a 3D-DS dimer, different intramolecular contacts that stabilize the monomeric structure must be broken and then recruited in an intermolecular fashion in the dimer. Since the residue pairs that participate in these quaternary contacts resemble those constituting monomeric contacts within a single polypeptide chain, there is no need to optimize a specific surface in an ancestral monomer to contact the partner subunit in the context of 3D-DS [9]. As a consequence, the only structural difference between a subunit in the 3D-DS dimer and its monomeric counterpart is the conformation of the hinge region, which connects the exchanging part in the intertwined dimer with the rest of its corresponding polypeptide chain [11] (Figure 1A).

Evidence of the evolutionary emergence of quaternary structures via 3D-DS in extant proteins is exemplary illustrated by histones. These are dimeric interleaved helical protein bundles where each monomer adopts a histone fold, common to all core eukaryotic histones and also present in archaeal histones and composed by two contiguous helix–strand–helix motifs connected as a result of a tandem duplication [12]. A sequence-based search for distant homologs employing Hidden Markov Models led to the identification of other protein folds also containing tandem duplications of these helix–strand–helix motifs, such as the C-domain of AAA+ ATPase proteins [13]. However, the C-domain is topologically different to the histone dimer in that it involves the association of the two consecutive motifs in a single polypeptide chain to constitute a four-helix bundle instead of protein–protein interactions between two monomers [13], which is a description that is consistent with the differences between isolated monomers and 3D-DS dimers.

Although no extant monomeric histones exist in nature, direct experimental evidence of the role of 3D-DS in the emergence of the dimeric histone fold was provided by adding a three-residue “insertion” (Gly-Thr-Pro), which is located in the middle of what otherwise would be the long helix in the histone fold of the C-domain of *Escherichia coli* RuvB, within the hinge region of the archaeal HMfB histone. This insertion disrupted the dimerization of HMfB and generated a soluble and stable monomer with properties consistent with a four-helix-bundle protein [14]. These results supported the idea that the dimeric histone fold originated through domain-swapping of two four-helix bundle monomers [14], thus providing compelling evidence of 3D-DS as a plausible evolutionary pathway for the origin of oligomeric structures in nature.

## 2. Molecular Evolution towards 3D-DS in Human FoxP Transcription Factors

While 3D-DS offers a simple evolutionary pathway to the emergence of oligomerization that does not require a specialized protein–protein interface, it is accompanied by the biophysical intricacy of requiring the breakage of many intramolecular contacts through at least partial protein unfolding to form an intertwined dimer [15]. Since the first description of 3D-DS in diphtheria toxin [9], seminal cases such as cyanovirin [16] and the yeast cell cycle controller p13suc1 [17] have shown that dimerization via 3D-DS is dramatically accelerated when these proteins are exposed to structural perturbations, i.e., pH and temperature changes or the addition of chemical denaturing agents, which is followed by restoring physiological conditions while employing high protein concentrations [18]. These observations demonstrate that reaching the unfolded state is a main limiting step to adopting the dimer for most studied cases. Moreover, the need of protein unfolding to speed up 3D-DS obscures the elucidation of the physiological significance of these intertwined oligomers [15].

Strikingly, a recent example of 3D-DS under physiological conditions has been observed in the FoxP subfamily of transcription factors. Fox proteins are present in yeast and metazoans, and several evolutionary analyses have strongly suggested that these proteins were present in the ancestor of all eukaryotes [19,20]. Their absence in plants suggests that their origin is linked to a clade of unicellular organisms that gave rise to both the fungal and animal lineages [21]. Moreover, evolutionary analyses combined with gene expression studies in the ancient invertebrate chordate amphioxus have demonstrated that a cluster of four Fox genes with sequential and coordinated endo-mesodermal tissue expression has been present since basal Bilateria and maintained in several lineages during animal evolution for more than 500 million years [22].

Recent bioinformatic, functional, and developmental analyses to identify and characterize the tissue-specific expression of different Fox proteins in representative organisms from the Metazoa branch have described a total of 25 Fox subfamilies, from A to S [23]. This increased diversity is reflected in the number of Fox proteins in different lineages from this branch, with mammalian genomes presenting at least 34 distinct Fox orthologs whereas only four genes are present in fungi [19]. In this regard, it has been suggested that the expansion of this protein family is the result of several duplications followed by loss and diversification events through multiple metazoan lineages during evolution [20]. This is the case for the emergence of the FoxS subfamily in the mammal and bird lineage divergence due to a duplication of a Fox gene from the original cluster present in basal Bilateria [24], which comprises the FoxC, FoxF, FoxL1, and FoxQ1 subfamilies [22].

Despite this diversification, Fox transcription factors can be grouped together based on the highly conserved structure and sequence of their ≈100 residue DNA-binding domain (DBD), also known as the forkhead domain [23] (Figure 1A). The DBD of Fox transcription factors folds into a canonical winged helix motif, which is composed of five helices (*H*1, *H*2, *H*3, *H*4, and *H*5), three β sheets (*S*1, *S*2, and *S*3) and two wings (*W*1 and *W*2) [25] (Figure 1A). Interestingly, this fold is also present in linker histones [23], which bind to and stabilize the nucleosome particle and participate in higher-order chromatin structures [26] but have a different evolutionary origin than core histones [27].

According to most solved Fox structures to date, their DBDs exist as monomers with helix *H*3 contacting the major groove of DNA [25,28,29,30,31]. While a canonical sequence RYAAAYA located on promoter regions has been defined as the target for Fox DBDs, phylogenetic and functional DNA-binding studies among the family found that some DBDs can bind to alternative DNA sequence motifs in addition to the canonical ones [32]. These results showed that changes in DNA-binding specificity across the Fox family were not explained by changes in DNA-contacting amino acids that define the specificity for canonical DNA binding sites. Moreover, some Fox proteins can specifically bind two sequence motifs. To explain these results, Nakagawa et al. [32] proposed that the DBD could adopt an alternative conformation with respect to the one observed in solved structures, allowing the recognition of additional DNA binding motifs. These insights suggest that structural heterogeneity may play a crucial role in the function of Fox proteins.

Regardless of the sequence and structure similarity between Fox proteins, they are differentially involved in several regulatory networks related to proliferation, differentiation, angiogenesis, apoptosis, and cell cycle progression [33]. Moreover, Fox subfamilies such as FoxC, FoxM, FoxP, and FoxA have been closely related to cancer [33,34]. Indeed, FoxP members have been described as oncogenes or tumor suppressors depending on cellular contexts [35], revealing the complexity of transcriptional networks in which these proteins are involved.

While the functional divergence between Fox proteins has been largely explained in terms of specific amino acid changes in the DBD throughout their evolution, and thus based on changes in binding affinity to their cognate DNA, these transcription factors also exhibit differences in terms of the presence or absence of accessory domains. For example, FoxP members are characterized by containing a trans repressor domain (TRD), a zinc finger domain (ZFD), and a leucine zipper domain (LZD), which are exclusively found in this subfamily [33] (Figure 1A). This multidomain heterogeneity could offer new aspects of functional regulation, such as interdomain communication and functional synergy.

Although the Fox family of transcription factors has been described as monomers even in the presence of their cognate DNA [30,36,37,38,39], suggesting their full functionality without requiring oligomerization, members of the FoxP subfamily show both monomers and 3D-DS dimers (Figure 1B) [40,41,42,43,44]. This novel ability in a well-known monomeric family has been largely attributed to a single replacement of a conserved proline by alanine (Pro39Ala) in the hinge region that connects helices *H*2 and *H*4 (Figure 1B). Additionally, the ability to adopt intertwined dimers has been a focus of interest in terms of the possibility to bind different DNA loci within a given chromosome or even in physically mediating interchromosomal contacts [45], suggesting that the emergence of the 3D-DS could impact their mechanism of action and the complexity of the gene regulation networks in which they participate.

As previously found in different studies, the reversion of the Pro-to-Ala evolutionary substitution in FoxP2 and FoxP1 abolished the generation of the dimeric population [40,42,43], suggesting that this single mutation was necessary to evolve to a 3D-DS dimer. Interestingly, other Fox members, such as FoxE3 [46], also show alanine in the same region, although biophysical studies are further required to determine their potential oligomerization via 3D-DS. However, several findings highlight the complex pathway by which 3D-DS emerged in this family. For example, the Pro39Ala mutation in the hinge region of FoxK1 was unable to generate the dimer [42], suggesting that more intricate sequence adaptations are needed. Moreover, a destabilizing triple mutant of FoxP3 including the aforementioned Ala-to-Pro reversal mutation could not completely abolish the dimerization in this protein [41], expanding the view regarding the sequence changes that modulate 3D-DS in the FoxP subfamily.

In this scenario, the analysis of sidechain contacts in the solved 3D-DS structure of FoxP2 identified a network of aromatic residues that stands out among the dimeric interface [40]. This network comprises residues Phe7, Tyr9, Trp33, Phe34, Phe38, Tyr40, Phe41, and Trp48 from both chains in the dimer [40]. All these residues are strictly conserved in the FoxP subfamily with the sole exception of Tyr40, which in FoxP3 is replaced by Phe. Interestingly, the Tyr40Phe mutant in FoxP2 decreased ≈10-fold its dissociation constant [44], thus favoring the dimeric form. In addition, sequence comparison shows that specific conserved residues in all FoxP members such as Phe7 and Phe34 from the aromatic network are replaced by other hydrophobic residues such as Tyr or Ile in the equivalent position in the rest of Fox sequences. Although this sequence change seems to be conservative, the lesson from mutant Tyr40Phe in FoxP2 suggests that minute but specific changes in biochemical properties of the sidechains in the hydrophobic network of the protein–protein interface can significantly impact the dimerization propensity.

In addition to the aforementioned relevant residues in the FoxP subfamily, other interesting molecular adaptations are observed when comparing FoxP members with the rest of the Fox family. In this context, Medina et al. showed that a specific histidine only conserved in FoxM, FoxO, and FoxP members and located in helix *H*3 can also modulate 3D-DS (Figure 1B) [47]. Different pH values around the pKa of that residue were used to analyze the effect of its (de)protonation in the dimerization and stability properties of FoxP1. Dimer dissociation experiments showed that the protonation of His59 increases the energetic barrier between the monomer and dimer, therefore hindering oligomerization. Likewise, equilibrium unfolding experiments and molecular dynamics showed that this protonation also decreases the global and local stability of FoxP1 by interrupting specific sidechain–backbone interactions between the imidazole group and a near-peptide bond located in the same helix. Hence, these residues in the FoxP family seem to complement the evolutionary Pro-to-Ala mutation in the hinge region to favor the emergence of 3D-DS dimers.

In light of the mutational, structural, and biophysical characterizations discussed above, the sequence changes occurring in the DBD of FoxP transcription factors complement the key evolutionary Pro-to-Ala substitution in the hinge region of the FoxP subfamily to enable this association process, beyond the proposition of 3D-DS as an ancestral association mechanism that does not require a specialized surface. It is worth noting that such amino acid substitutions in so-called secondary interfaces, which only exist in the 3D-DS conformation and thus contribute to its propensity by stabilizing the dimer, have been proposed in previous works [18].

## 3. Biophysically Dissecting the Evolutionary Strategies of FoxP Proteins to Overcome the Thermodynamic Limitations of 3D-DS

As mentioned above, 3D-DS emerged in the FoxP subfamily as an evolutionary novelty to promote protein association. However, most of the studied 3D-DS models to date conclude that the acquisition of the intertwined dimer (or oligomer) is kinetically limited by protein unfolding [16,17,18,48]. Although this behavior has been widely observed in canonical examples of 3D-DS [48,49,50], the human FoxP subfamily has shown different kinetic and thermodynamic properties when compared with such models [40,41,42,43,47].

For FoxP proteins, most of the thermodynamic and quantitative information regarding 3D-DS has been extracted from FoxP1. Chu et al. [42] determined that the equilibrium dissociation constant of human FoxP1 at 37 °C is 27 µM, which is at least 100-fold lower than well-characterized models such as p13suc1 [17], cyanovirin-C [16], and cytochrome *c* 552 [51]. In addition, a series of dissociation equilibrium experiments in a range of temperatures from 20 to 37 °C performed by Medina et al. [43] determined the temperature-dependence of the dimer dissociation constant for FoxP1, ranging between 1 and 30 µM. These results support the fact that the dimerization of FoxP1 requires low protein concentrations compared with most of the canonical 3D-DS models. Furthermore, both works showed that low protein concentrations are enough to reach the FoxP1 3D-DS equilibrium in minutes at 37 °C instead of hours or days (or even longer times). In this case, the dissociation is disfavored by 2–3 kcal·mol^−1^ when compared to canonical models of 3D-DS such as p13suc1, cyanovirin-c, and RNase S [16,17,52]. All these results highlight the importance of determining the structural and biophysical properties that distinguish FoxP proteins from the canonical thermodynamic and kinetics aspects of 3D-DS.

One of the main hypotheses that explains the properties shown by FoxP1 is a decrease in protein stability. Specifically, the decrease in the energy barrier that imposes the loss of native contacts in FoxP1 could be compensated by the intermolecular stabilization of the structure once the dimer is adopted [53,54,55,56]. In this line, NMR analysis of the monomeric and dimeric states of FoxP1 indicated that helices *H*2 and *H*3, and the wing *W*1 are notably flexible compared with the other secondary structures in the chain [42]. Thus, stability changes could promote at least local unfolding. In addition, these analyses determined that the dimer is more flexible throughout its backbone when compared with the monomer, giving preliminary clues about the role of structural flexibility in the stability and propensity of 3D-DS.

Medina et al. [43] further analyzed the relationship between stability and flexibility in FoxP1 by comparing the wild-type protein with the Ala39Pro mutant that is unable to dimerize [42,43]. Equilibrium unfolding experiments determined the presence of a native-like monomeric intermediate with high secondary structure content that is accumulated when wild-type FoxP1 is incubated with mild-denaturing conditions, suggesting that 3D-DS is coupled with local unfolding. This behavior is absent in the monomeric mutant, indicating that the folding mechanism of FoxP1 is strongly influenced by 3D-DS and that the presence of this locally-unfolded intermediate could decrease the kinetic limitations of this association [43]. Moreover, these experiments also evidenced that the monomeric mutant is ≈2 kcal·mol^−1^ more stable than wild-type FoxP1, supporting the relation between protein stability and 3D-DS. These results agree with similar studies performed in other models of 3D-DS [54,57,58,59,60], highlighting the idea that the decrease in stability of the native state is an evolutionary strategy to favor the dimerization event.

**Figure 2:**
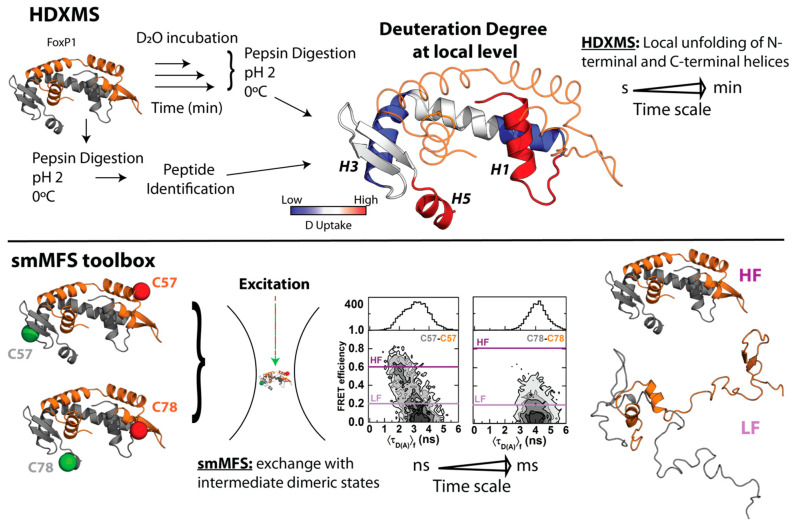
Insights into the 3D-DS pathway in FoxP1. Using high-resolution experimental and computational approaches, the mechanism by which FoxP1 dimerizes via 3D-DS was proposed. By using HDXMS, differences in flexibility between secondary structure elements of the protein suggested that helices *H*1 and *H*5 and strand *S*1 are prone to unfold in the monomer (in red), facilitating the accumulation of the monomeric intermediate described in equilibrium unfolding experiments. To detect these differences, the protein is first incubated in deuterated buffer (D_2_O) at different times (from seconds to few minutes); then, the reactions are quenched, and lastly, the sample is pepsin-digested, and all identified peptides are analyzed by mass spectrometry. In addition, a smMFS toolbox was used to describe the local dynamics of the double-labeled 3D-DS dimer at the single-molecule level under pulsed interleaved excitation (PIE) in a confocal volume, finding the presence of the predicted folded dimer (High-FRET state, HF) and the accumulation of a dimeric intermediate ensemble that is locally unfolded (Low FRET, LF). As an example, we show the results from two different FRET pairs (C57–C57 and C78–C78), which monitor the dynamics between helices *H*3 of the dimer, or between helices *H*3 and *H*5. The differences between FRET populations in both sets of FRET pairs indicates the differences in structural behavior of different regions of the same protein. By comparing the measured FRET distances with those intermediate structures observed in folding molecular dynamics simulations, a structural view of the mechanism of 3D-DS in FoxP can be inferred. The time scales for HDXMS and smMFS are shown to describe their temporal resolution.

In order to gain more insights regarding local structural changes in FoxP1 upon 3D-DS, hydrogen–deuterium exchange coupled with mass spectrometry (HDXMS) was used. This experimental strategy ascertains the solvent accessibility and/or flexibility of local regions of a given protein through its incubation in a deuterated buffer, such that the extent of deuteron incorporation from the solvent into the backbone amides of a protein acts as a mass probe, which is followed by its proteolytic digestion and final analysis by mass spectrometry to monitor these changes over local protein regions (Figure 2) [61,62]. In the case of FoxP1, HDXMS experiments on the wild-type protein under physiological and mild-denaturing conditions described regions with high flexibility (as determined by their higher deuteron incorporation when compared to other local regions within the protein), such as helices *H*1 and *H*5, and beta strand *S*1 (Figure 2), suggesting the relevance of these localized regions for both the monomeric intermediate acquisition as well as favoring the dimerization of FoxP1 [43]. Moreover, the comparison between FoxP1 and the mutant Ala39Pro showed a stabilization of the region *H*1-*S*1 that could be a relevant factor to modulate 3D-DS in FoxP proteins, further strengthening that local rather than global structural perturbations suffice to facilitate their dimerization.

Altogether, these experiments represent the first indications of an evolutive strategy in this subfamily to overcome the thermodynamic limitations of 3D-DS. A decrease in protein stability and an increase in local structural flexibility seemed to be correlated with the dimerization (and dissociation) properties of FoxP proteins. However, none of those approaches explain how these structural features relate with the mechanism by which FoxP associates via 3D-DS.

## 4. The Molecular Mechanism of 3D-DS Explained at Near-Atomic Level

For FoxP proteins, flexibility and stability are relevant to understand the molecular mechanism by which they dimerize. This connection is not unknown, considering that proteins are constantly fluctuating, and their local or even global dynamics can be strongly influenced by intra- and intermolecular interactions, and vice versa [63]. Thus, dissecting protein dynamics must consider structural heterogeneity at the proper timescales.

In the case of FoxP1, single-molecule Multiparameter Fluorescence Spectroscopy (smMFS) toolbox and HDXMS were employed to elucidate the molecular pathway of 3D-DS and the relationship with structural dynamics at different timescales [64]. These approaches were used considering their powerful temporal and structural resolution that are informative from fast structural motions [65,66] as well as their effects in order–disorder transitions taking place within the dimerization timescales (Figure 2). The smMFS toolbox takes into account fluorescent methodologies such as Förster Resonance Energy Transfer (FRET), Fluorescence Correlation Spectroscopy (FCS), and molecular dynamics to monitor conformational changes in the nanosecond to millisecond timescale at near-atomic level [65,66]. For HDXMS, deuteron incorporation kinetics from seconds to minutes allow the identification of changes over the dimerization timescales observed for FoxP1 [67,68].

When analyzed at short timescales with smMFS in native conditions, FoxP1 behaves as a system in which the 3D-DS dimer dynamically exchanges with an extended and dimeric intermediate ensemble in a microseconds to milliseconds timescale. In this approach, single-cysteine mutants were labeled with donor (D) or acceptor (A) dyes for FRET to combine between them, allowing the monitoring of the structural dynamics of different regions of the domain-swapped dimer. As an example, we show two FRET dimers, where one of them was generated by labeling of S57C in both monomers (C57(D)-C57(A)) and the other was generated through labelling of V78C in both monomers (C78(D)-C78(A)) (Figure 2). These experiments demonstrated that different secondary structure elements in FoxP1 are more likely to remain folded in the 3D-DS conformation or in the intermediate ensemble, although extreme cases such as helices *H*1 and *H*5 are mostly in the extended ensemble, suggesting that they are primarily found as disordered elements (Figure 2) [64]. Additionally, native-centric molecular dynamics simulations of FoxP1 using structure-based models that enable simulating folding and binding transitions [69] determined that small energy barriers separate the 3D-DS dimer and the intermediate ensemble, thus providing a thermodynamic explanation based on order–disorder transitions to the dimerization properties of FoxP1.

The kinetic approximation of HDXMS was important to detect a large amount of deuteron incorporation throughout the polypeptide chain (≈30–60%) within the timescales in which FoxP1 dimerization occurs, without significant differences between monomers and dimers except that the *H*2–*H*4 helix that is rearranged as an extended helix in the 3D-DS ensemble (Figure 1B) [64]. The monitoring of FoxP1 also showed that helix *H*5 exhibits the largest extent of deuteron incorporation (≈50–60%), as previously deduced from smMFS. Furthermore, deuteron incorporation kinetics from helix *H*1 showed two different populations of fast and slowly exchanging amides (inferred from their biphasic exponential pattern), with an increase in the number of backbone amides exhibiting slow deuteron incorporation under mild denaturing conditions, while no significant changes were observed in the number of fast amides. This behavior indicates that stable amides unable to exchange in native conditions are now more available to deuteration. These results suggest that this helix could be prone to major structural changes upon dimerization or even local unfolding under native conditions.

In summary, structural changes of FoxP1 within a broad timescale from microseconds to minutes allows the accumulation of dimeric intermediate ensembles with a large fraction of highly flexible and disordered elements. These order–disorder transitions dramatically contribute to facilitating 3D-DS in FoxP proteins and put forward that these traits in protein dynamics are specifically encoded within this subfamily during the evolution of the Fox family of transcription factors.

## 5. Evolution Pathway inside FoxP Subfamily and Their Impact in Functionality: From Homodimers to Heterodimers and Beyond

While FoxP1 is the most biophysically characterized member in the subfamily, these observations could be described as general features for all FoxP members given that the DBDs of these transcription factors exhibit a high degree of sequence identity (75–92%).

**Figure 3:**
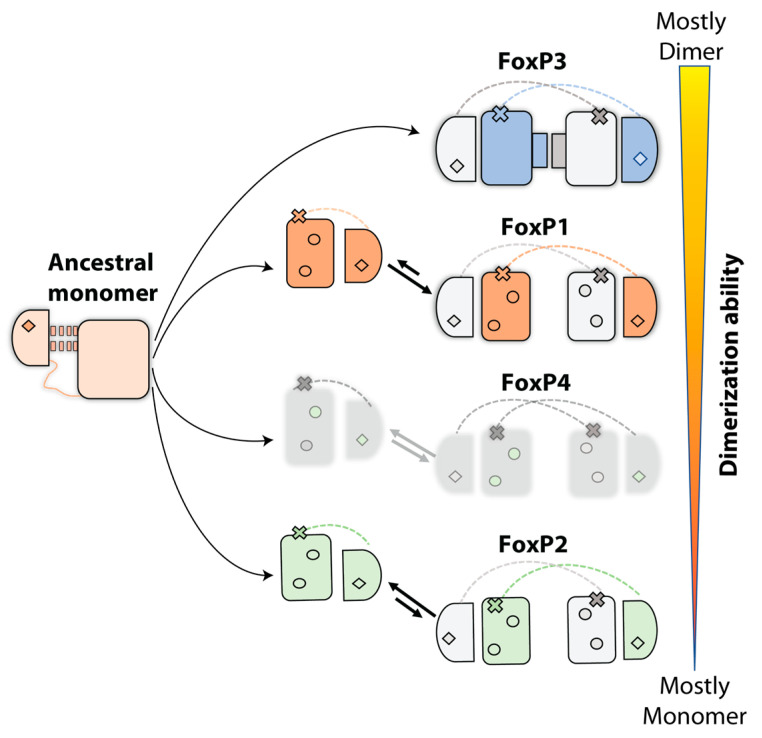
Proposed 3D-DS evolution pathway within the Fox family. An ancestral monomer accumulated non-deleterious random mutations (rhombus) before the emergence of 3D-DS in the Fox family. Inside this group, a hydrophobic network that stabilizes the dimerization interface could be considered as residues that favor dimerization, but they are insufficient to enable dimerization by themselves. The conserved Ala39Pro mutation (x) in the hinge region allowed this ancestral monomer to generate 3D-DS dimers within the FoxP subfamily. All residue substitutions from the monomeric ancestor (rhombus) are conserved in the FoxP subfamily, although FoxP3 contains some sequence differences that allow an additional stabilization of the interface formed by helices *H*1 and *H*2 (small rectangles). FoxP4, a still uncharacterized member of this subfamily, is shown in gray, but its sequence similarity with respect to FoxP1 and FoxP2 suggests that it could have a dimerization propensity within the protein concentration range defined by the dimer dissociation constants of these proteins.

Nevertheless, while a similar mechanism for 3D-DS is expected among FoxP proteins, there is extensive evidence that minimal sequence changes effectively impact each monomer–dimer equilibrium. For example, FoxP1 and FoxP2 show notable differences in their reported dissociation constant in spite of sharing 89% of sequence identity in their DBDs [40,43,44,47]. For the still uncharacterized FoxP4, the sequence comparison with FoxP1 and FoxP2 shows 92 and 89% of identity, respectively, suggesting that FoxP4 could have a dissociation constant between the reported values of FoxP1 and FoxP2 (Figure 3). However, the lack of biophysical data for this protein limits our understanding regarding its 3D-DS properties. Lastly, FoxP3 has been characterized as an obligated dimer of the subfamily [41] and exhibits the lowest sequence identity with the rest of the FoxP subfamily, ranging from 75% to 80%. Thus, it seems that FoxP3 has differentially evolved from the rest of the FoxP subfamily members toward the formation of a secondary interface in its 3D-DS dimer (Figure 3).

The sequence comparison of the DBDs from all human FoxP shows that changes are heterogeneously distributed along the primary structure (Figure 1B). However, a large proportion of them are located in regions between helix *H*1 and the hinge loop. FoxP3 has the most striking sequence changes, since amino acids substitutions observed in this protein replace biochemical properties of their counterparts in the rest of the FoxP subfamily. Structure comparison between FoxP2 and FoxP3 suggests that 3D-DS properties are explained by the presence of a secondary dimerization interface of FoxP3 [41], which compacts the dimer of FoxP3 when compared to FoxP2, as it has been suggested by structural comparison of both proteins [41]. This interface is stabilized by contacts between both helices *H*1 from different polypeptide chains and their respective helices *H*2. Experimental characterization of the quaternary state of FoxP3 double-mutant W16Q/M36T by SEC-MALS experiments, where the equivalent residues in FoxP2 were introduced in the secondary interface, resulted in the observation of a similar population of monomer and dimer [41]. Furthermore, a triple mutant that also included the Ala39Pro mutation could not completely abolish dimerization, which is in notable contrast with the observed behavior in FoxP2 [40,44] and FoxP1 [43].

An evolutionary pathway forming a secondary interface to enhance intersubunit contacts in a 3D-DS dimer was suggested as the primitive mechanism to form stable intertwined dimers in IFN-y, IL-5, and βb2-crystallin [9]. Since those proteins do not have a monomeric structure, comparing their intertwined dimer with closely monomeric homologs structures showed that the interdomain interface of the dimer resembles the monomeric interdomain interface. Therefore, domain swapping could be the first step in the evolution of these dimers, which is followed by the formation of a secondary interface [9]. Considering the aforementioned higher dissociation constants and higher sequence identity between FoxP1, FoxP2, and FoxP4 when compared with FoxP3, the FoxP family constitutes an excellent model to dissect the mutations accumulated throughout evolution that provide primary and secondary interfaces for the emergence of 3D-DS and its specialization into obligated dimers, and to examine the different dimerization properties exhibited by these transcription factors in the context of their direct impacts on functional diversification.

Considering all these biophysical, structural, and evolutionary elements, the possibility to associate and generate heterodimers becomes relevant in terms of functional and structural complexity. In fact, FoxP1 is co-expressed in different scenarios with FoxP2 and FoxP4 in the brain [70], and it co-precipitates with the same protein partners [70,71]. Moreover, some relevant processes in mammals are dependent on the presence of FoxP1 and FoxP2 or FoxP4 [70,71,72], implying that some heteroassociation must be required to perform their cellular function.

Several computational studies have indicated that 3D-DS depends primarily on the monomer’s topology [53,55,73] and the structural characteristics of the hinge loop [17,52,74]. Experimental studies on covalently fused immunoglobulin domains with varying degrees of sequence identity from the muscle protein titin showed that their propensity of aggregation into dimers is high when the sequence identity is above 70% [75], such that low sequence identity is necessary to avoid misfolding. Similar studies at the single-molecule level and accompanied by native-centric molecular dynamics demonstrated that the interactions underlying misfolding in these domains are the result of sequence-specific 3D-DS [76]. Building upon these results, molecular dynamics simulations using force fields that take into account the sequence-dependence of protein interactions demonstrated that the reduction of the sequence identity between covalently linked multidomains decreased the formation of 3D-DS contacts, and that these contacts compete with other strong hydrophobic self-recognition contacts, leading to non-3D-DS misfolding in a balance that can be modulated by point mutations [77].

These insights suggest the possibility of heteroassociation via 3D-DS in the FoxP subfamily due to their high sequence identity. Although some studies showed the ability to heterodimerize between FoxP members [71,78], this process has not been biophysically explored, opening an interesting question about the functional and structural role of FoxP1 in increasing the association ability of the other FoxP members.

In addition, FoxP proteins are structurally more complex as they contain ZFD and LZD as accessory domains, which have been extensively studied as relevant in functions such as DNA-binding [79,80] and protein dimerization [81,82] in different eukaryotic transcription factors. These elements suggest a functional redundancy or even a synergy between domains FoxP proteins, with effects in their structural properties and therefore in their dimerization abilities. In fact, some studies with FoxP proteins have shown that the presence of LZD impacts not only the dimerization [83] and the DNA-binding processes [84] but also the ability to heterodimerize [71,78], highlighting the importance of these domains in the evolution of FoxP subfamily. Still, the specific structural characterization of FoxP proteins and their implication in their function needs to be deeply investigated.
